# Pulmonary neoplasia mimicking fungus ball

**DOI:** 10.1590/0100-3984.2015.0119

**Published:** 2015

**Authors:** Bruno Fernandes Cavalcante, Gláucia Zanetti, Edson Marchiori

**Affiliations:** 1 Department of Radiologiy - Universidade Federal do Rio de Janeiro (UFRJ), Rio de Janeiro, RJ, Brazil.

*Dear Editor,*

We report the case of a 74-year-old man smoking 80 cigarette packages per year, with history of pulmonary tuberculosis for 50 years. Two years ago, the patient underwent chest computed tomography that demonstrated centrilobular and paraseptal emphysema, besides sparse bullae, the largest one located in the right lower lobe, with a small nodular mass inside, measuring about 0.8 cm in diameter (Figure 1A).

**Figure 1:**
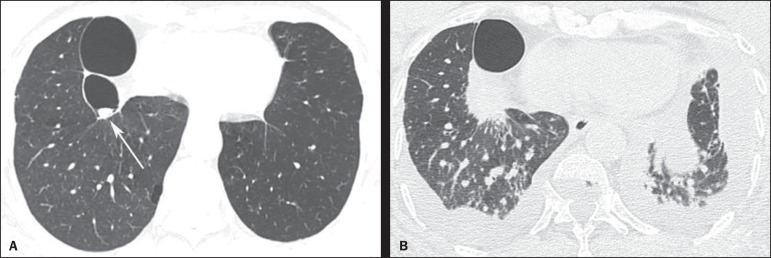
HRCT scan at the level of the lung bases (**A**) showing two bullae at right, with a small nodular mass measuring about 0.8 cm in diameter inside the small bulla (arrow). On **B**, scan acquired two years later, with a section of the same region, showing the presence of a mass with spiculated borders, adjacent to the posterior portion of the largest bulla, occupying the small bulla where the nodular mass had been seen at the previous CT images. Also, observe the presence of interstitial thickening suggestive of carcinomatous lymphangitis, besides bilateral pleural effusion.

The patient didn't return for follow-up and after two years presented with progressive dyspnea whose onset had occurred two months ago, in association with cough, weight loss and pain in the lower third of the right hemithorax. A new chest computed tomography demonstrated a mass with spiculated margins, adjacent to the posterior portion of the largest bulla, occupying the whole bulla where the nodular mass had been seen at the previous computed tomography images (Figure 1B). Also, interstitial thickening suggestive of carcinomatous lymphangitis was observed, besides bilateral pleural effusion.

Pericardium biopsy and cytological analysis of pleural effusion revealed adenocarcinoma, raising the hypothesis of lung adenocarcinoma with metastasis to the pleura and pericardium. A chemotherapy protocol with gemcitabine and carboplatin was initiated. The patient presented worsening of the respiratory condition, progressing to death after two months.

Lung cancer frequently presents like a nodule or solitary lung mass^(1,2)^. However, the disease presentation forms are quite variable and some typical findings may be observed. One of such findings is growth from a preexisting cystic mass, mimicking a fungus ball. Thus, a cystic image showing either focal or diffuse wall thickening progressing to a nodular mass should include lung tumor in the differential diagnosis^(3)^, particularly in cases where the nodule is attached to the wall and does not move with change in decubitus.

Other conditions which may present the finding of fungus ball include Rasmussen aneurysms, hydatid cysts, abscesses and intracavitary hematomas, besides fungal diseases themselves (aspergillosis, nocardiasis, actinomycosis, candidiasis, coccidioidomycosis)^(2,4)^.

As the neoplasm develops in previous pulmonary lesions, it is found especially in fibroatelectatic or granulomatous areas resulting from sequelae, generally associated with tuberculosis. The occurrence of lung cancer in cavities mimicking fungus ball or air crescent sign is quite rare^(1,2,5)^. The tumor tends to infiltrate in the adjacent pulmonary parenchyma causing a paracicatricial effect, and may lead to emphysematous or cystic changes adjacent to the neoplastic process^(1)^.

In conclusion, lung cancer must be considered in the differential diagnosis for patients who present with a fungus ball-like lesion, particularly in cases where the nodule is fixed to the cavity wall.

## References

[1] Wang LF, Chu H, Chen YM (2007). Adenocarcinoma of the lung presenting as a mycetoma with an air
crescent sign. Chest.

[2] Gazzoni FF, Severo LC, Marchiori E (2014). Pulmonary diseases with imaging findings mimicking
aspergilloma. Lung.

[3] Truong MT, Ko JP, Rossi SE (2014). Update in the evaluation of the solitary pulmonary
nodule. Radiographics.

[4] Watanabe H, Uruma T, Tsunoda T (2014). Lung metastasis of transitional cell cancer of the urothelium,
with fungus ball-like shadows closely resembling aspergilloma: a case report
and review of the literature. Oncol Lett.

[5] Bandoh S, Fujita J, Fukunaga Y (1999). Cavitary lung cancer with an aspergilloma-like
shadow. Lung Cancer.

